# Preparation of Polymer Electrolyte Membranes via Radiation-Induced Graft Copolymerization on Poly(ethylene-alt-tetrafluoroethylene) (ETFE) Using the Crosslinker *N*,*N*′-Methylenebis(acrylamide)

**DOI:** 10.3390/membranes8040102

**Published:** 2018-11-06

**Authors:** Xi Ke, Marco Drache, Uwe Gohs, Ulrich Kunz, Sabine Beuermann

**Affiliations:** 1 Institute of Technical Chemistry, Clausthal University of Technology, Arnold-Sommerfeld-Straße 4, 38678 Clausthal-Zellerfeld, Germany; xi.ke@tu-clausthal.de (X.K.); marco.drache@tu-clausthal.de (M.D.); 2 Leibniz Institute of Polymer Research, Hohe Straße 6, 01069 Dresden, Germany; gohs@ipfdd.de; 3 Institute of Chemical and Electrochemical Process Engineering, Clausthal University of Technology, Leibnizstraße 17, 38678 Clausthal-Zellerfeld, Germany; kunz@icvt.tu-clausthal.de; 4 Energie-Forschungszentrum Niedersachsen, Am Stollen 19A, 38640 Goslar, Germany

**Keywords:** polymer electrolyte membranes, ETFE, graft copolymerization, crosslinker, fuel cell, vanadium redox-flow battery

## Abstract

Polymer electrolyte membranes (PEM) prepared by radiation-induced graft copolymerization are investigated. For this purpose, commercial poly(ethylene-alt-tetrafluoroethylene) (ETFE) films were activated by electron beam treatment and subsequently grafted with the monomers glycidyl methacrylate (GMA), hydroxyethyl methacrylate (HEMA) and *N*,*N*′-methylenebis(acrylamide) (MBAA) as crosslinker. The target is to achieve a high degree of grafting (*DG*) and high proton conductivity. To evaluate the electrochemical performance, the PEMs were tested in a fuel cell and in a vanadium redox-flow battery (VRFB). High power densities of 134 mW∙cm^−2^ and 474 mW∙cm^−2^ were observed, respectively.

## 1. Introduction

With the gradual exhaustion of fossil fuels and a growing awareness of environmental protection, research and development in renewable energy and energy storage is being driven forward. In recent decades, the interests of the scientific community and industry have focused on fuel cell and redox-flow batteries (RFB) [1,2,3,4,5,6,7]. In terms of high efficiency, low greenhouse gas emissions and flexible deployment, polymer electrolyte membrane fuel cells (PEMFC) operated with H_2_/O_2_ are considered as a key technology [8,9,10,11]. As the vital component for fuel cell and vanadium redox-flow battery (VRFB), a polymer electrolyte membrane (PEM) should possess high proton conductivity, good mechanical stability, and selective ion transport [12,13,14,15,16]. Increased use of renewable energy sources has made the development of efficient energy storage systems more important in recent years [17,18,19]. The pumped storage power station is currently the world’s most widely used energy storage system. However, the high construction costs and the limited availability of suitable locations restrict the use of this technology [20]. The RFB systems are currently considered as potential alternative energy storage systems [21,22,23,24]. The advantages include, for example, unlimited capacity, high scalability, low construction costs and good operational reliability [25,26]. On the other hand, the RFB has a relatively low energy density, so the RFB is not optimal to use in the mobile domain [27].

Key components in PEMFCs and in vanadium RFB are PEM. Two types of PEMFCs are considered, depending on the operating temperature: high temperature and low temperature fuel cells. Frequently, PEMs are synthesized via a radiation-induced graft polymerization on a base material [28,29,30]. One example are strategies employing a commercial poly(ethylene-alt-tetrafluoroethylene) (ETFE) base material. Previously, we reported on high temperature polymer electrolyte membranes (HTPEM) based on induced graft copolymerization of (meth)acrylate monomers on ETFE backbone material after electron beam (EB) treatment and subsequent doping with phosphoric acid [31,32]. In contrast, generally PEMs with an operating temperature below 100 °C (low temperature polymer electrolyte membranes, LTPEM) contain sulfonic acid groups, which are nowadays most commonly used in fuel cell vehicles. One representative is the commercial Nafion membrane [33,34] with a high proton conductivity. Nafion is a sulfonated tetrafluoroethylene-based fluoropolymer and shows excellent thermal, mechanical, and chemical stability. It is used in fuel cell applications and in vanadium redox-flow batteries (VRFB). However, the high costs of Nafion membranes often obstruct widespread industrial application [35,36,37,38]. Therefore, an alternative membrane to replace Nafion is needed.

Grafting reactions require the “activation” of polymer molecules, which can be achieved by chemical or physical methods. In the case of EB, this activation is independent of temperature, molecular structure of the polymer and state of aggregate. In contrast to chemical initiators, the use of EB irradiation is preferred in a swollen polymer medium due to exact control of temperature. EB technology is used in various industrial applications for crosslinking, grafting, polymerization, degradation, and functionalization of polymeric materials for more than 50 years [39]. Free radicals are generated by spatial and temporal precise absorption of electron energy without any use of chemical additives. The process is easily to handle, cost and time saving. The advantage of producing PEM with the electron beam-induced graft polymerization is that the ETFE backbone is already present as a film. There are no further process steps required for membrane production. The monomers used in the graft polymerization are inexpensive and readily available since they are also used for other applications, e.g., for coatings, and become proton-conducting by sulfonation and a high functional density of proton-conducting sulfonic acid groups can be achieved.

In this contribution a synthetic approach is reported, which combines the EB activation of a commercial ETFE foil and subsequent radical copolymerization of glycidyl methacrylate (GMA) and hydroxyethyl methacrylate (HEMA) [40]. To improve the properties of the polymer membranes, especially the Coulomb efficiency in VRFB, in exploratory work it was shown that the addition of 1,4-butanediol diacrylate as crosslinker led to a significant reduction in crossover [40]. Previous PEM synthesis using radiated ETFE was rather slow with a polymerization time of seven hours [41]. After EB activation of the ETFE membrane with a dose of 100 kGy the solvent content selected was 60% and the reaction temperature was set to 60 °C. This avoids the formation of significant amounts of polymer in the reaction mixture, which is not grafted to the backbone material [42,43,44,45]. To reduce the polymerization time, it appeared important to decrease the solvent content in the polymerization mixture and to enhance the polymerization temperature. In addition, the ETFE base material was activated with a lower dose ranging from 40 to 60 kGy to reduce the density of trapped radicals. It was anticipated that due to the lower density of trapped radicals, fewer side reactions inside the reaction mixture happen and that less termination occurs, resulting in longer grafted polymer chains. To gain further information on the impact of crosslinker addition on PEM synthesis and on its properties an alternate crosslinker was tested. The feasibility of PEMs obtained with the improved polymerization procedure and *N*,*N*′-methylenebis(acrylamide) MBAA as crosslinker for application in a low temperature fuel cell and in a VRFB is reported.

## 2. Experimental

### 2.1. Materials

Poly(ethylene-alt-tetrafluoroethylene) (ETFE) film (ET-film 6235Z, Nowoflon, Siegsdorf, Germany) with a thickness of 50 μm was used. Glycidyl methacrylate (GMA, ≥97%, Aldrich, Steinheim, Germany) and hydroxyethyl methacrylate (HEMA, 97%, Aldrich, Steinheim, Germany) were purified using an inhibitor remover column (Aldrich, St. Louis, MO, USA) and stored at 4 °C in the dark before use. *N*,*N*′-methylenebis(acrylamide) (MBAA, 99%, Aldrich, St. Louis, MO, USA), dimethylformamide (DMF, ≥97%,Alfa Aesar, Kandel, Germany), sodium bisulfite (Acros, Geel, Belgium), sodium sulfite (96%, Aldrich, Steinheim, Germany), 2-sulfobenzoic acid anhydride (SBA, 94%, Alfa Aesar, Kandel, Germany) und sulfuric acid (99%, Merck, Darmstadt, Germany) were used as received.

### 2.2. Membrane Synthesis

#### 2.2.1. Activation of the Films

The ETFE films were cut to a size of 10 cm × 10 cm and packed in polyethylene bags. The EB treatment of the base films (1.5 MeV, 4 mA) was carried out with an electron accelerator (type ELV-2, BINP Novosibirsk) at the Leibniz Institut für Polymerforschung Dresden e.V. The dose amounted to 40, 50 and 60 kGy. The irradiated membranes were stored for a maximum period of 6 months at a temperature of −30 °C until use. Polymer radicals are trapped at temperatures below the glass transition temperature due to low polymer chain segment mobility and in the crystalline domains. Therefore, there is no significant loss of radicals through radical combination for a long period of time. For further details refer to [41].

#### 2.2.2. Radiation-Induced Graft Polymerization

In analogy to previous reports [40,46,47,48,49] the graft copolymerization was carried out in a 100 mL double walled glass reactor equipped with a condenser and a gas inlet to allow for purging with nitrogen to remove oxygen from the reaction mixture. Additionally, the content of the reactor is mixed by the gas flow. The monomer feed consists of GMA (85 mol %), HEMA (10 mol %), and the crosslinker MBAA (5 mol %). DMF is used as solvent with solvent fractions discussed below. The monomers and DMF were added to the reactor, the mixture was purged with nitrogen for 10 min, and then heated to the reaction temperature. Subsequently, the membrane (5 cm × 5 cm) was added to the reactor as quickly as possible. For further exclusion of oxygen, continuous purging with nitrogen was applied. After the reaction, the sample was placed in 100 mL pure DMF overnight to extract the excess monomer from the surface. Thereafter, the membrane was washed 3 times with 50 mL methanol and dried to a constant mass. The degree of grafting (*DG*) is the determining measure for quantifying the growth of the polymer membrane. It can be calculated as follows.

(1) $$DG\;=\;\frac{m_{p}{-m}_{0}}{m_{0}}\times 100\%\;$$

where *m*_p_ and *m*_0_ are the masses of the membrane before and after the graft reaction, respectively.

#### 2.2.3. Sulfonation

**Scheme 1:**
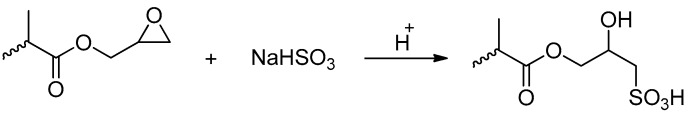
Sulfonation reaction of GMA.

**Scheme 2:**
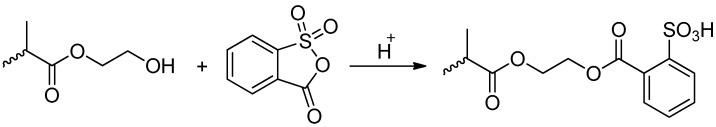
Sulfonation reaction of HEMA.

For the sulfonation of GMA, a mixture of sodium sulfite, sodium bisulfite, isopropanol, and water in the ratio 10/3/10/77 wt % was prepared. In 100 mL of this mixture the grafted membrane was heated for 6 h at 70 °C and subsequently washed with 500 mL distilled water. HEMA was sulfonated with 100 mL of a 0.2 M solution of SBA in the solvent DMF at 70 °C. The sulfonated membrane was then washed several times with distilled water and protonated for one day in 0.5 M sulfuric acid. The sulfonation reactions of both monomers GMA and HEMA are illustrated in Scheme 1 and Scheme 2. For further information, the reader is referred to reference 40 and 49.

### 2.3. Membrane Analysis

#### 2.3.1. Fourier-Transformed Infrared Spectroscopy (FTIR)

For FTIR analyses a Vertex 70 spectrometer (Bruker Optik GmbH, Ettlingen, Germany) was employed using a globar source and a photoacoustic cell (PA301). The FTIR spectra were recorded in the wavenumber range from 400 cm^−1^ to 4000 cm^−1^ with a resolution of 4 cm^−1^ and 20 scans.

#### 2.3.2. Electrochemical Impedance Spectroscopy (EIS)

The specific conductivity of the PEM was determined using an IviumStat EIS instrument (Ivium Technologies, Eindhoven, The Netherlands). To prepare the measuring cell, the membrane was clamped between two stainless steel electrodes with a defined torque of 0.5 Nm. The measurements were carried out in a water bath at temperatures between 30 °C and 60 °C with temperature increments of 10 K within the frequency range from 1 MHz to 10 Hz. This covers approximately the temperature range of operation of the fuel cell. According to Equation (2), the specific conductivity is calculated:

(2) $$\sigma (\mathrm{mS}\cdot {\mathrm{cm}}^{-1})=\frac{d_{M}}{A\cdot {(R-R}_{0})}$$

- *d*_M_: thickness of the membrane (cm)
- *A*: area of the electrode (cm^2^)
- *R*: total resistance with the membrane (mΩ)
- *R*_0_: total resistance without the membrane (mΩ)

#### 2.3.3. Ion Exchange Capacity (IEC) and Water Uptake (WU)

To determine the concentration of sulfonic acid groups in the PEM, an acid-base titration was carried out with 0.005 mol·L^−1^ H_2_SO_4_ solution. The dry PEM (2 cm × 3 cm) was kept in 100 mL 0.1 mol·L^−1^ NaOH solution for 2 days. The sulfonic acid groups in the PEM can react with the base completely. For comparison, a blank solution without PEM was prepared under the same conditions. 10 mL of the NaOH solution from the sample with PEM and the blank solution are pipetted in a beaker with 5 drops indicator solution (Tashiro: 0.1 g methyl red and 0.05 g methylene blue in 100 mL ethanol). The standard solution (0.005 mol·L^−1^ H_2_SO_4_ solution) was added drop by drop in the alkaline solution with constant swirling of the beaker until the color changes from green to violet. According to Equation (3), IEC of the PEMs were calculated.

(3) $$IEC=\frac{B-P}{m}\;\times 0.01\times 10\;$$

where *B* and *P* are the added titrant volume of H_2_SO_4_ solution for the blank solution and the sample with PEM, respectively, while *m* is the mass of the dry PEM.

Water uptake describes the water retention capacity of the PEM. This value can be calculated using Equation (4)

(4) $$WU=\frac{m_{\mathrm{wet}}-m_{\mathrm{dry}}}{m_{\mathrm{dry}}}$$

where, *m*_wet_ and *m*_dry_ are the mass of the wet and dry PEMs, respectively.

#### 2.3.4. Fuel Cell Test

The fuel cell measurements were carried out in the temperature range from 30 °C to 50 °C with a stoichiometric gas excess ratio of 1.1 for both gases (λ_H2_, anode = 1.1, λ_O2_, cathode = 1.1). λ_H2_ is defined as the ratio of the amount of H_2_ fed to the cell and the amount of H_2_ that reacted. λ_O2_ is defined accordingly for O_2_. To control the volume flow of hydrogen and oxygen, two rotameters (Fischer & Porter) were used. An electrochemical load (Höcherl & Hackl, Konzell, Germany) was used to control the applied current density and voltage.

The membranes were tested in a single fuel cell with an active area of 5 cm × 5 cm. The key component is the membrane electrode assembly (MEA), which comprises of a PEM with an area of 6 cm × 6 cm, two gas diffusion electrodes (GDE, BASF Fuel Cell HT251EWSI) with a Pt loading of 0.5 mg·cm^−2^ and two Kapton films (DuPont, Wilmington, DE, USA) to fix the set-up. Two extra gas diffusion layers (GDL, Freudenberg H2315 I3 C1) were inserted between the MEA and bipolar plates of the cell to reach a good electrical contact [47,50]. Before the actual start of the test, each membrane was conditioned at a voltage of 0.1 V and variable current density. For further details refer to [41]. Thereafter, the electrochemical performance of the PEM is generally stable. For the fuel cell test, polarization curves were recorded in the current density range from 4 mA·cm^−2^ to 480 mA·cm^−2^. The ohmic resistance is determined in the range from 120 mA·cm^−2^ to 320 mA·cm^−2^. Continuous measurements were performed to investigate the power density and power stability. Continuous measurements were carried out at a current density of 200 mA·cm^−2^ for five hours. Power densities were determined at this operating point.

#### 2.3.5. Mechanical Properties

The mechanical stability of the polymer membranes is determined with a Zwick Z2.5/TN1S instrument. The tested membranes were cut in pieces with a width of 10 mm and a length of 100 mm. The measurements were run at a speed of 10 mm·min^−1^ with a preload of 0.2 N·mm^−2^. The tensile tests are carried out at least three times.

#### 2.3.6. VRFB

The membrane was tested in a 10 cm^2^ VRFB with 150 mL electrolyte of 1.6 mol·L^−1^ V (50% VO^2+^ and 50% V^3+^) in 4 mol·L^−1^ H_2_SO_4_ (GfE Metalle und Materialien GmbH, Nürnberg, Germany) in both tanks [40]. The membrane was measured with 10 charge and discharge cycles (CDC) and two polarization curves (before and after the CDC) at room temperature. As a reference, Nafion 117 was tested in the same way. The Coulomb efficiency was calculated using Equation (5):

(5) $$\mathrm{Coulomb}\;\mathrm{efficiency}\;=\;\frac{\mathrm{discharge}\;\mathrm{capacity}\;(\mathrm{Ah})}{\mathrm{charge}\;\mathrm{capacity}\;(\mathrm{Ah})}\;$$

For the polarization curve, the measurement starts with a charged battery state, which means 100% state of charge (SoC) and shows an open cell voltage of approximately 1.65 V. The vanadium electrolyte is pumped at a flow rate of 40 mL·min^−1^ through the anode and the cathode. After every 10 s, the current density increased in steps of 28.5 mA·cm^−2^ until a voltage of 0.2 V is reached. The voltages measured at varied current density yield the polarization curve.

## 3. Results and Discussion

### 3.1. Membrane Synthesis

For membrane synthesis, the main influencing parameters of the graft polymerization were systematically investigated to achieve the highest target variable (here: grafting degree). Therefore, experiments were planned and carried out according to the principles of the design of experiments (DOE). In this case, three parameters were varied, for each three values were chosen: the reaction temperature, *x*_1_, (60 °C, 80 °C, 100 °C), the solvent fraction, *x*_2_, (40%, 50%, 60%) and the dose, *x*_3_, (40 kGy, 50 kGy, 60 kGy). In total 27 experiments were carried out. For further details on the DOE please refer to the Supplementary Materials.

Initially, the reaction time for these experiments was set to three hours. However, it turned out that this time is not suited for all experiments. Generally, the reaction time is limited due to the risk of polymer formation in the solution because of chain transfer to the monomers and the solvent, which is associated with a strong increase in viscosity. Since the reaction mixture inside the polymerization reaction turns into a gel, this process is called gelling [42,43,44,45]. To avoid gelling at higher reaction temperatures the time had to be reduced. In addition, low solvent fractions may afford lower times. For the polymerizations carried out in the presence of the crosslinker the consequences of polymerization in the solution are more severe: due to crosslinking of the growing chains the viscosity increase is stronger since larger macromolecules may be formed. The higher the reaction temperature the more the reaction time must be reduced to avoid gelling. The actual reaction times ranging from 75 to 180 min will be discussed below.

**Figure 1:**
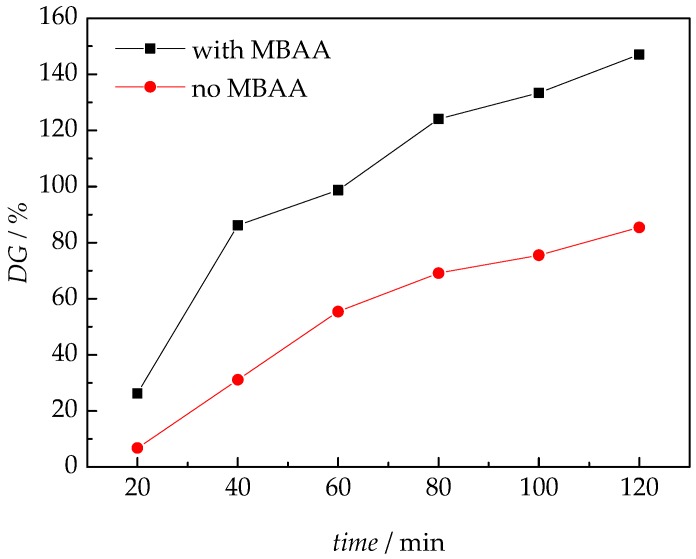
Influence of the crosslinker on the development of the *DG* with time for *x*_1_ = 80 °C, *x*_2_ = 40 vol %, *x*_3_ = 50 kGy with 85 mol % GMA, 10 mol % HEMA, and 5 mol % MBAA.

To gain a better understanding of the variation of grafting degree (*DG*) with reaction time, in addition to the DOE further experiments were carried out. The goal was to investigate the influence of crosslinker, the correlation between reaction temperature and monomer content, and to identify the best reaction time. Figure 1 shows the evolution of *DG* (*x*_1_ = 80 °C, *x*_2_ = 40%, *x*_3_ = 50 kGy) with time in the presence and the absence of crosslinker. The reaction time was set to a maximum of two hours to minimize the risk of polymer formation in the solution. One series of polymerizations was performed without the crosslinker MBAA, a second series with MBAA being present in the reaction mixture. The data in Figure 1 indicates that the grafting degree of membranes from polymerizations in the presence of MBAA are significantly higher than that in the absence of MBAA. After two hours a *DG* of 150% is reached in the presence of MBAA, whereas only a *DG* of around 85% is obtained in the absence of the crosslinker. Generally, acrylate monomers have significantly higher propagation rate coefficients than methacryaltes and the polymerization proceeds much faster [51]. Thus, the acrylate-type crosslinker is preferably incorporated into the copolymer.

**Figure 2:**
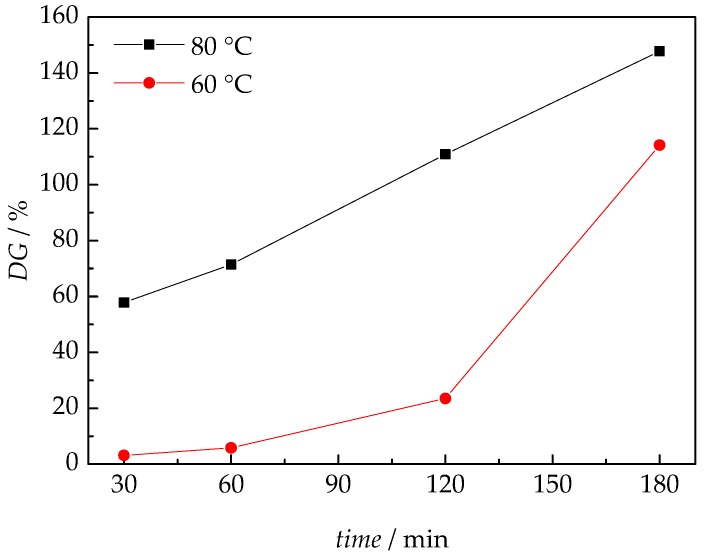
Influence of reaction temperature on the *DG* with *x*_2_ = 40 vol %, *x*_3_ = 40 kGy, 85 mol % GMA, 10 mol % HEMA, and 5 mol % MBAA.

The strong influence of the reaction temperature on the *DG* is illustrated in Figure 2. For both temperatures, membranes activated with 40 kGy were used. The solvent volume fraction is 60% in the polymerization mixture. At 60 °C only minor grafting degrees were obtained in the first two hours. However, within the third hour of the reaction, there is a sudden increase in *DG* to a maximum value of around 115%. In contrast, at 80 °C already after 30 min a *DG* of 57% is reached. With increasing time, a steady increase in *DG* is observed leading to a value of 147% after three hours. Due to formation of polymer in the solutions a prolongation of reaction time was not feasible.

As stated above a reaction temperature of 80 °C is associated with the problem of gelling, which limits the reaction time. Thus, it appeared rewarding to perform additional experiments not contained in the original DOE, in which the monomer content in the solution was reduced to 20% and 30% by volume. The dose applied to the base film is 40 kGy. *DG* levels of the grafted membrane for a monomer content of 20 vol % and 30 vol % in the reaction mixture are only 20% and 43%, respectively. Despite lowering the monomer content still significant amounts of polymer were formed in solution and the viscosity increased. Accordingly, a lowering of the monomer content for better control of the reaction at 100 °C is not expected to be effective. As a consequence, experiments at 100 °C were not performed.

**Figure 3:**
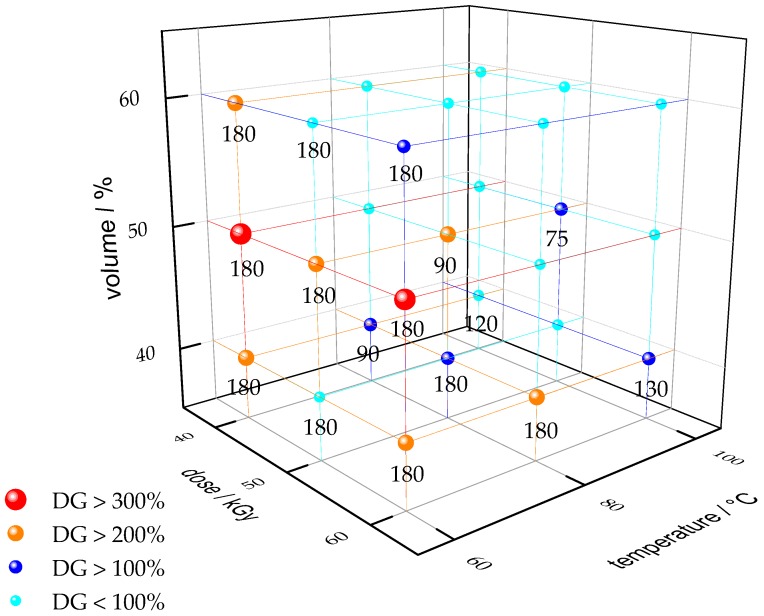
Impact of the influencing variables (reaction temperature, solvent content, and irradiation dose) from the design of experiments on the degree of grafting *DG* for polymerizations with a monomer feed consisting of 85 mol % GMA, 10 mol % HEMA, and 5 mol % MBAA. The numbers correspond to the reaction time in minutes.

The results from the DOE presented in detail in the supporting information are summarized in Figure 3. The numbers inside the diagram refer to the reaction times in minutes. The size and color of the markers indicate the *DG* reached. It is clearly seen that the highest degrees of grafting, higher than 300%, are obtained at 60 °C, which may be suggested to be the optimum reaction temperature for the synthesis of crosslinked ETFE-*g*-P(GMA-*co*-HEMA). The highest *DG* of 392% was obtained for (*x*_1_ = 60 °C, *x*_2_ = 50%, *x*_3_ = 60 kGy) in 180 min. Another interesting point is *x*_1_ = 80 °C, *x*_2_ = 50% and *x*_3_ = 50 kGy, because already in 90 min a *DG* of 230% is achieved. Previously, it was shown that a PEM with *DG* of 230% may compete with the electrochemical properties of a Nafion 117 membrane in fuel cells or VRFB [40,41].

At first sight it might have been anticipated that a lowering of the dose and the associated reduction in concentration of trapped radicals is not favorable for the PEM synthesis. However, due to the lower density of trapped radicals inside the base material it was possible to increase the monomer content in the reaction mixture and to enhance the polymerization temperature. These two changes more than compensate a lowering of the polymerization rate due to lower density of trapped radicals in the membrane material and the reaction times may be reduced by more than a factor of two compared to a dose of 100 kGy. In addition, a lower concentration of radicals is associated with a lower termination probability and the graft lengths are expected to be larger, which is identified by the high values for *DG*.

### 3.2. Analyses

Generally, membranes with high grafting degrees are associated with a large number of functional groups on the grafted membrane. Thus, these membranes are expected to possess a good conductivity. However, a high grafting degree also means a low content of the backbone material, which is associated with a decreasing mechanical stability of the grafted membrane. For further testing two PEMs were selected. PEM #2 with a high *DG* of 284% was characterized via FTIR and EIS as well as tested in a low temperature fuel cell. This polymer membrane has both a high grafting degree and a smooth surface, which is suitable for use in the fuel cell. #2 was synthesized with *x*_1_ = 60 °C, *x*_2_ = 40%, *x*_3_ = 40 kGy. For comparison, a membrane (DG225) synthesized in the absence of crosslinker with almost identical *DG* of 225% is analyzed. PEM #5 with a *DG* of 230% was used for the VRFB test.

#### 3.2.1. Fourier-Transformed Infrared Spectroscopy (FTIR)

**Figure 4:**
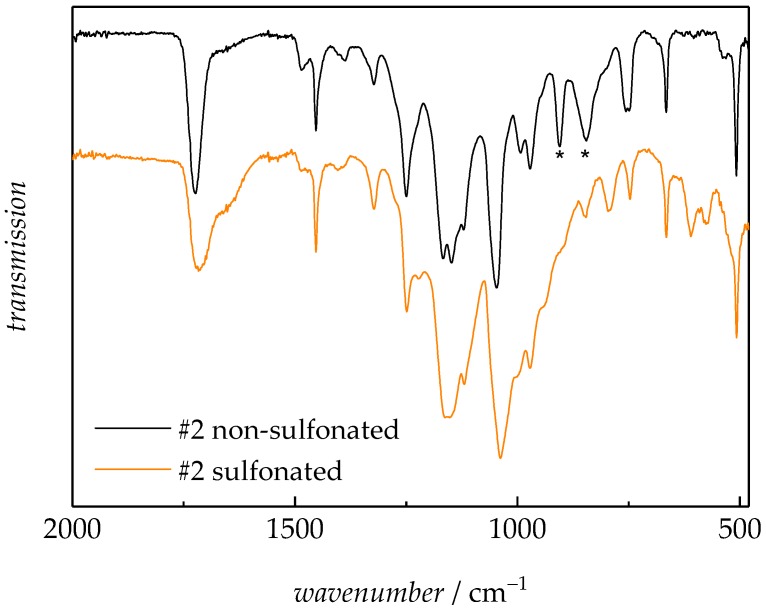
IR spectra for sample #2 before (black) and after (orange) sulfonation of GMA units. * mark absorptions due to the epoxy group.

Figure 4 shows the FTIR spectra of sample #2 before and after sulfonation. The epoxide group of the GMA units is associated with two signals at 905 cm^−1^ and 854 cm^−1^, marked by the asterisks [52]. As indicated by the spectra, both peaks are reduced in intensity after sulfonation of the GMA moieties in the grafted membrane.

#### 3.2.2. EIS

**Figure 5:**
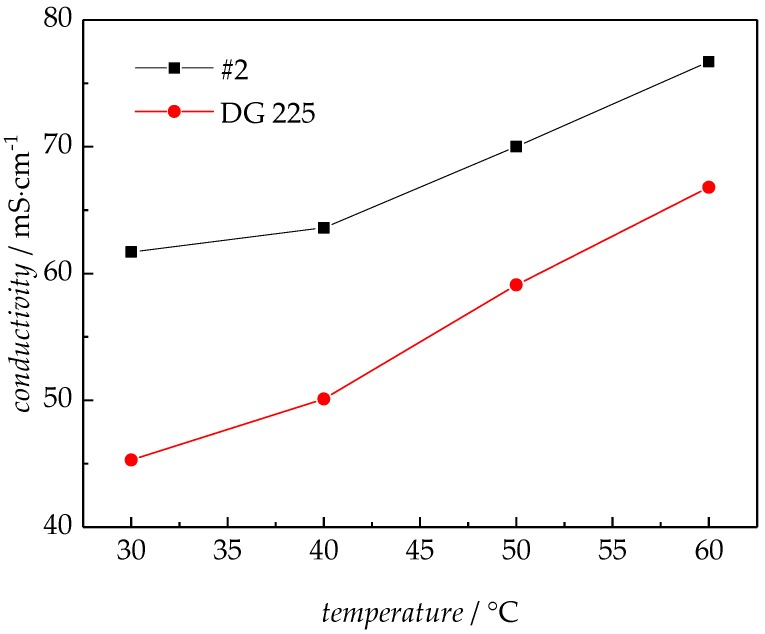
Conductivity in the range of the operating temperature for fuel cell and vanadium redox-flow battery.

The electrical conductivity of the membranes after sulfonation was measured by EIS according to the method described above for the temperature range from 30 °C to 60 °C in steps of 10 K. The results are given in Figure 5. In addition, data for membrane DG225 synthesized without crosslinker is depicted. An increase in conductivity with temperature is clearly seen. It is obvious that the crosslinked membrane #2 shows a higher conductivity. The expected increase in conductivity with temperature is due to a higher mobility of ions at higher temperatures. However, it must be considered that *DG* of #2 is 284%, which contributes to the higher conductivity, too.

High conductivity is a prerequisite for the usability of PEM in technical facilities. Proton conduction takes place due to both, the sulfonic acid groups, and water. Therefore, a successful sulfonation of the functional groups of the grafted membrane is essential.

#### 3.2.3. Fuel Cell Test

**Figure 6:**
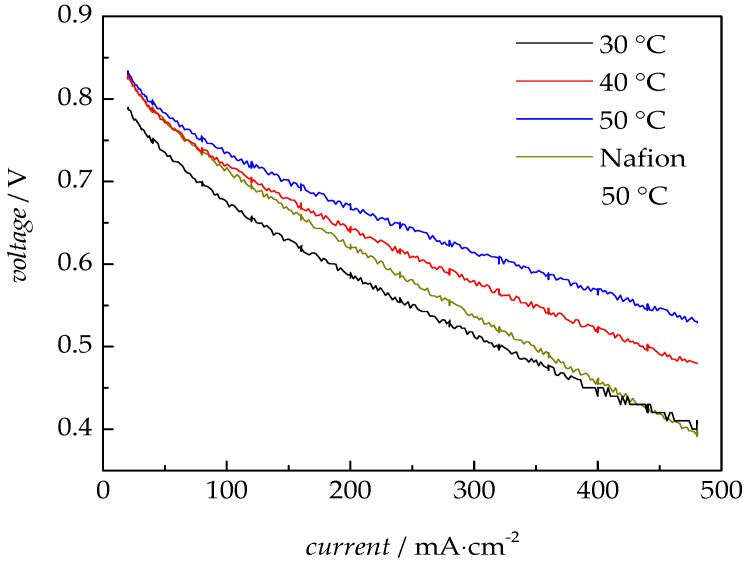
Polarization curves of sample #2 measured in a fuel cell from 30 to 50 °C after being kept at a current of 200 mA·cm^−2^ for five hours. For reference data for Nafion 117 measured at 50 °C is included.

The sulfonated PEM #2 was tested in the H_2_/O_2_ fuel cell. At each operating temperature, the membrane stayed inside the cell at a current density of 200 mA·cm^−2^ for five hours. After each continuous 5 h measurement a polarization curve was recorded. The polarization curves are shown in Figure 6.

**Figure 7:**
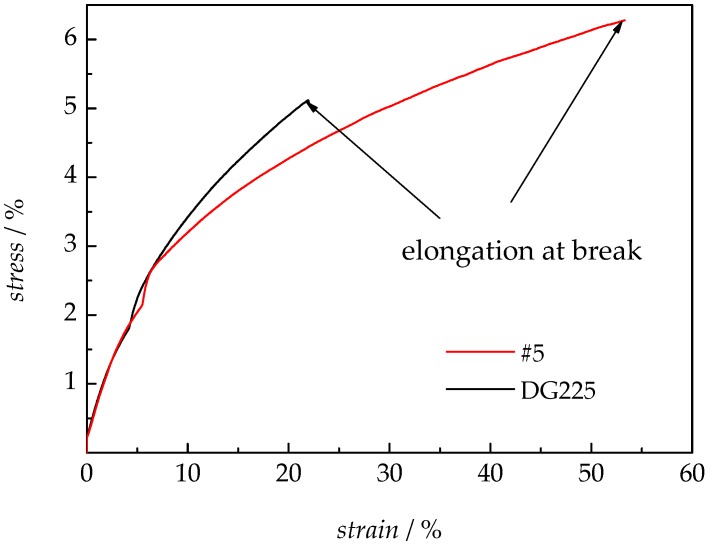
Mechanical stability of the crosslinked PEM #5 and a PEM synthesized without crosslinker (DG225).

The open-circuit electrode potential of the fuel cell is temperature independent at 0.98 V. The non-linear region of the activation polarization is observed up to about 120 mA·cm^−2^, after that the linear ohmic region is seen, in which the internal resistance of the membranes in Table 1 was determined. Even at high current levels (point measurements to 600 mA·cm^−2^), the gas supplied in the cell is sufficiently high (lambda 1.1) and the polarization curve remains linear. The ohmic resistances, *R*_Ω_, are determined from the slope of the curves in the range between 120 mA∙cm^−2^ and 320 mA∙cm^−2^. As shown in Figure 7, the resistance of the PEM decreases with increasing operating temperature from 30 °C to 50 °C. At 50 °C, the PEM indicates a very low internal resistance of 21 mΩ. The non-crosslinked PEM with a *DG* of 225% (DG225) has an ohmic resistance of 22 mΩ at 50 °C, indicating that crosslinking has a negligible impact on the resistance. Under the same conditions, the resistance of a Nafion 117 membrane was measured to be 34 mΩ [41]. This finding is similar to the results of EIS. Obviously, in comparison to the Nafion 117 membrane, the synthesized polymer membrane has a lower resistance. The performance of the PEM in a fuel cell is summarized in Table 1.

For sample #2, the IEC of 2.89 meq∙g^−1^ and the water uptake of 223% at room temperature were additionally determined. Nafion has a much lower IEC of 0.9 meq∙g^−1^ [53]. This also explains the better performance of sample #2 in the fuel cell compared to Nafion.

#### 3.2.4. Mechanical Analysis

To avoid the cross over effect, the crosslinked membrane #5 (*x*_3_ = 80 °C, *x*_2_ = 50%, *x*_3_ = 50 kGy) with a relatively high *DG* of 230% was tested in a VRFB. Before the battery test, the mechanical stability of the crosslinked membrane was measured by the tensile test. ETFE itself is mechanically very stable and has a high value of elongation at break (195%). This parameter for mechanical stability decreases with increasing *DG* [40]. In addition, the polymer membrane DG225 synthesized without any crosslinker was used as reference. The results are shown in Figure 7. Apparently, the use of crosslinker has a positive influence on the membrane’s stability. Compared to the membrane without crosslinker, the elongation at break is doubled for the crosslinked membrane. An elongation at break of 53% is reached. Tensile strength (*R*_m_), modulus of elasticity (*E*) and elongation at break (*ε*-break) are listed in Table 2.

#### 3.2.5. VRFB Tests

**Figure 8:**
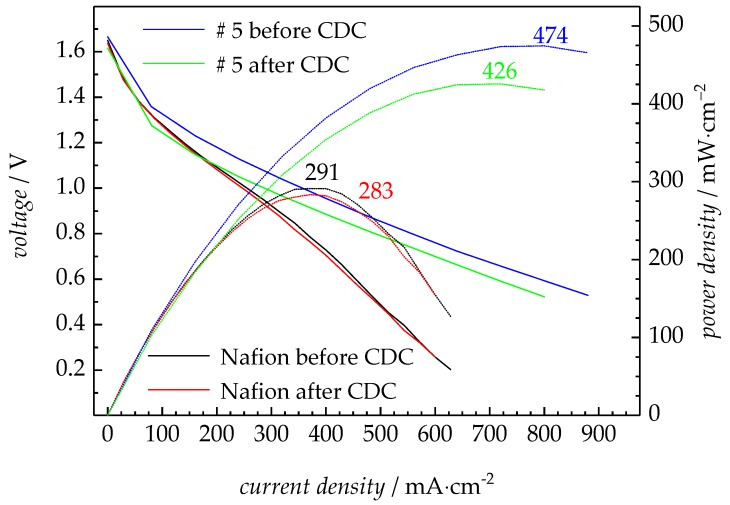
Polarization curves of sample #5 and Nafion 117. The numbers indicate the power density with the units of mW∙cm^−2^.

The mechanical stability of the membrane #5 is sufficient for a VRFB test. 10 CDC were performed. Polarization curves were measured before and after the CDC. The results are shown in Figure 8. For comparison corresponding data for Nafion 117 are included. The data indicates that the crosslinked polymer membrane performs well in the VRFB system. Compared to Nafion 117 an excellent maximum power density with more than 400 mW∙cm^−2^ is observed. The corresponding value for Nafion is below 300 mW∙cm^−2^. Sample #5 has a high IEC of 2.24 meq∙g^−1^ and a water uptake of 194% at room temperature resulting in high conductivity in the VRFB. For both membranes, the voltage is lower for all current densities after carrying out 10 CDCs. The maximum power density decreases by 10% for sample #5 and 3% for the Nafion 117 membrane.

The crossover effect changes the vanadium concentration in both electrolyte tanks and leads to a reduced performance of the battery. To limit the impact of the differences in the electrolyte reservoirs on the polarization curves, for the measurement with PEM #5 the electrolytes from both tanks were balanced after the CDC, but the electrolyte was not replaced. The open cell voltage for the test of #5 decreases from 1.66 V before to 1.62 V after the CDCs. The slightly reduced voltage may be due to the used electrolyte. In addition, the slope of the polarization curve after 10 CDCs is not changed, indicating that the membrane was not damaged in the process. The reference measurements of the polarization curves with Nafion 117 before and after 10 CDCs were carried out with a fresh electrolyte. The differences in the polarization curves and the power densities are even less pronounced. 

**Figure 9:**
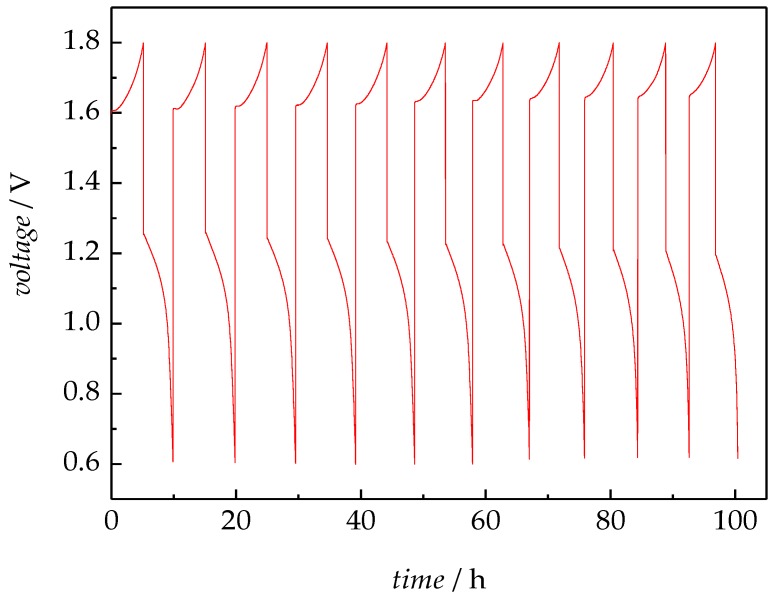
10 charge and discharge cycles for #5.

**Figure 10:**
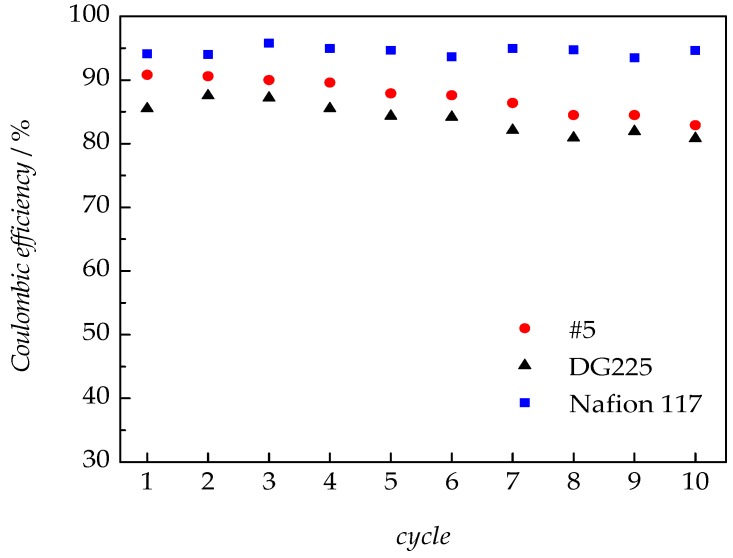
Coulomb efficiency of the sample #5, DG225 and Nafion.

Figure 9 shows that the voltage of the cell in the charged and discharged state remains at the same level over the 10 CDCs. The first CDC takes about 10 h, which is slightly reduced for the last cycle. The shorter the time of one CDC the lower the capacity of the battery. The crossover effect of the PEM in the battery test is particularly well described by the Coulomb efficiencies given by Equation (3), which is the ratio of the charge and discharge capacity. Points in Figure 10 are calculated from the cycles shown in Figure 9. Nafion 117 and a polymer membrane without crosslinker (DG225) serve as references.

The results show that the Coulomb efficiency of the membrane with crosslinker (#5) is higher than that of the membrane without crosslinker (DG225) over the entire range. This finding may be interpreted that the crosslinked membrane shows a reduced crossover effect. However, both membranes are below the level of Nafion 117. With the high grafting degree, the polymer membrane has grown large after the reaction. It leads to the higher crossover effect compared to Nafion. The improvement of the Coulomb efficiency observed for the membrane synthesized with the crosslinker MBAA is similar to the previously published results [40], where 1, 4-butanediol diacrylate was used as crosslinker. The major difference in the here presented new PEM syntheses is that significantly lower doses and lower amounts of solvent were used, which lead to less side reactions in the free volume of the polymerization reactor and a higher mechanical stability of the membranes. Moreover, the reaction times are significantly shorter. These aspects can contribute to a more effective membrane production process.

## 4. Conclusions

PEMs for the use in a H_2_/O_2_ fuel cell and a VRFB were synthesized and tested. An important parameter for the performance of the membranes is the content of grafted polymer (degree of grafting, *DG*), which is directly responsible for the functional density of the sulfonic acid. In contrast to previous work rather low dose between 40 kGy and 60 kGy were used. The goal was to lower the risk of side reactions. It was anticipated that an increase of the monomer fraction and the temperature may lead to an enhanced grafting rate. The DOE approach was used to investigate the influence of several reaction parameters, such as temperature, solvent content, and dose, on *DG*. Generally, the reaction time is greatly reduced in the presence of a crosslinker. 60 °C is a rather robust reaction temperature to produce the crosslinked PEM, allowing for reaction times of 3 h. At 80 °C with a solvent fraction of 50% and a dose of 50 kGy a suitable DG is already obtained in 90 min.

To evaluate the electrochemical performance of the membranes, two PEMs synthesized were selected for feasibility tests in a fuel cell and a VRFB. Membrane #2 with a *DG* of 284% was chosen for fuel cell test and membrane #5 with a *DG* of 230% for VRFB. The crosslinked PEMs have a very high power density both in fuel cell and VRFB tests. In the fuel cell test at 50 °C, a constant power density of 134 mW∙cm^−2^ is achieved at a current density of 200 mA∙cm^−2^. In a VRFB test the crosslinked and the non-crosslinked membrane show a lower Coulomb efficiency in comparison to a Nafion 117 membrane due to the high grafting degree, with the crossover effect of the membrane being reduced for the crosslinked membrane. In addition, an excellent power density of 474 mW∙cm^−2^ is observed for the crosslinked PEM, much higher than for Nafion, which reached only 291 mW∙cm^−2^. With the electron beam-induced graft polymerization, a targeted adjustment of the properties according to the application in fuel cells or VRFBs is possible. Major influencing factors are the *DG* and the additional use of crosslinkers.

## Figures and Tables

**Table 1:** Performance and the resistance of the PEM in the fuel cell test.

Sample	Power Density after 5 h at 200 mA∙cm^−2^/mW∙cm^−2^	Resistance/mΩ
#2 at 30 °C	118	26
#2 at 40 °C	128	24
#2 at 50 °C	134	21
DG225 at 50 °C [41]	129	22
Nafion 117 at 50 °C [41]	124	34

**Table 2:** Results of tensile strain measurements for the crosslinked PEM #5 and the polymer membrane DG225.

Sample	*R*_m_/N∙mm^−2^	*E*/N∙mm^−2^	*ε*-Break/%
**#5**	6.28	43.3	53.3
**DG225**	5.12	49.9	22.0

## Supplementary Materials

The following are available online at http://www.mdpi.com/2077-0375/8/4/102/s1, Figure S1: Three-dimensional representation of the experimental design, Figure S2: Applied reaction times in minutes with appropriate DG in the experimental design, Table S1: Starting point (xio) and the step size (dxi) of the influencing variables, Table S2: Points of the experimental design and target value DG, Table S3: Additional points to the experimental plan and target value DG.

## References

[1] Chalk S.G., Miller J.F., Wagner F.W. (2000). Challenges for fuel cells in transport applications. J. Power Sources.

[2] Chu D., Jiang R., Gardner K., Jacobs R., Schmidt J., Quakenbush T., Stephens J. (2001). Polymer electrolyte membrane fuel cells for communication applications. J. Power Sources.

[3] Lee K., Kang S., Ahn K. (2017). Development of a highly efficient solid oxide fuel cell system. Appl. Energy.

[4] Tang A., Ting S., Bao J., Skyllas-Kazacos M. (2012). Thermal modelling and simulation of the all-vanadium redox flow battery. J. Power Sources.

[5] Prifti H., Parasuraman A., Winardi S., Lim T.M., Skyllas-Kazacos M. (2012). Membranes for redox flow battery applications. Membranes.

[6] Tang A., Bao J., Skyllas-Kazacos M. (2014). Studies on pressure losses and flow rate optimization in vanadium redox flow battery. J. Power Sources.

[7] Li X., Xiong J., Tang A., Qin Y., Liu J., Yan C. (2018). Investigation of the use of electrolyte viscosity for online state–of–charge monitoring design in vanadium redox flow battery. Appl. Energy.

[8] Wang Y., Long W., Wang L., Yuan R., Ignaszak A., Fang B., Wilkinson D.P. (2018). Unlocking the door to highly active ORR catalysts for PEMFC applications: Polyhedron-engineered Pt-based nanocrystals. Energy Environ. Sci..

[9] Saghali Z., Mahmoudimehr J. (2017). Superiority of a novel conic tubular PEM fuel cell over the conventional cylindrical one. Int. J. Hydrog. Energy.

[10] Ding Z., He L., Dong Z., Gao X. (2013). An integrated numerical model for a PEM fuel cell system. Adv. Mater. Res..

[11] Chutichai B., Authayanun S., Assabumrungrat S., Arpornwichanop A. (2013). Performance analysis of an integrated biomass gasification and PEMFC (proton exchange membrane fuel cell) system: Hydrogen and power generation. Energy.

[12] Rao Z., Zheng C., Geng F. (2018). Proton conduction of fuel cell polymer membranes: Molecular dynamics simulation. Comput. Mater. Sci..

[13] Junoh H., Jaafar J., Norddin M.N.A.M., Ismail A.F., Othman M.H.D., Rahman M.A., Yusof N., Salleh W.N.W., Ilbeygi H. (2014). A review on the fabrication of electrospun polymer electrolyte membrane for direct methanol fuel cell. J. Nanomater..

[14] Parasuraman A., Lim T.M., Menictas C., Skylla–Kazacos M. (2013). Review of material research and development for vanadium redox flow battery applications. Electrochim. Acta.

[15] Luo T., David O., Gendel Y., Wessling M. (2016). Porous poly(benzimidazole) membrane for all vanadium redox flow battery. J. Power Sources.

[16] Li J., Yuan X., Liu S., He Z., Zhou Z., Li A. (2017). A low-cost and high-performance sulfonated polyimide proton-conductive membrane for vanadium redox flow/static batteries. ACS Appl. Mater. Interfaces.

[17] Bhattacharjee A., Roy A., Banerjee N., Patra S., Saha H. (2018). Precision dynamic equivalent circuit model of a vanadium redox flow battery and determination of circuit parameters for its optimal performance in renewable energy applications. J. Power Sources.

[18] Huang Y., Zheng Y., Li X., Adams F., Luo W., Huang Y., Hu L. (2018). Electrode materials of sodium–ion batteries toward practical application. ACS Energy Lett..

[19] Wu X., Leonard D.P., Ji X. (2017). Emerging non-aqueous potassium-ion batteries: Challenges and opportunities. Chem. Mater..

[20] Aalianvari A. (2014). Optimum depth of grout curtain around pumped storage power cavern based on geological conditions. Bull. Eng. Geol. Environ..

[21] Zhao Y., Ding Y., Li Y., Peng L., Byon H.R., Goodenough J.B., Yu G. (2015). A chemistry and material perspective on lithium redox flow batteries towards high-density electrical energy storage. Chem. Soc. Rev..

[22] Soloveichik G.L. (2015). Flow batteries: Current status and trends. Chem. Rev..

[23] Noack J., Roznyatovskaya N., Herr T., Fischer P. (2015). The chemistry of redox-flow batteries. Angew. Chem. Int. Ed..

[24] Winsberg J., Hagemann T., Janoschka T., Hager M.D., Schubert U.S. (2017). Redox–flow batteries: From metals to organic redox-active materials. Angew. Chem. Int. Ed..

[25] Wang W., Luo Q., Wei X., Li L., Yang Z. (2013). Recent progress in redox flow battery research and development. Adv. Funct. Mater..

[26] Skyllas-Kazacos M., Cao L., Kazacos M., Kausar N., Mousa A. (2016). Vanadium electrolyte studies for the vanadium redox battery—A review. ChemSusChem.

[27] Li Z., Weng G., Zou Q., Cong G., Lu Y. (2016). A high-energy and low-cost polysulfide/iodide redox flow battery. Nano Energy.

[28] Gubler L. (2014). Polymer design strategies for radiation-grafted fuel cell membranes. Adv. Energy. Mater..

[29] Nasef M.M. (2014). Radiation-grafted membranes for polymer electrolyte fuel cells: Current trends and future directions. Chem. Rev..

[30] Kraytsberg A., Ein-Eli Y. (2014). Review of Advanced materials for proton exchange membrane fuel cells. Energy Fuels.

[31] Li X., Drache M., Gohs U., Beuermann S. (2015). Novel concept of polymer electrolyte membranes for high-temperature fuel cells based on ETFE grafted with neutral acrylic monomers. J. Membr. Sci..

[32] Li X., Drache M., Ke X., Gohs U., Beuermann S. (2016). Fuel cell application of high temperature polymer electrolyte membranes obtained by graft copolymerization of acrylic acid and 2-Hydroxyethylmethacrylate on ETFE backbone material. Macromol. Mater. Eng..

[33] Luo Z., Chang Z., Zhang Y., Liu Z., Li J. (2010). Electro–osmotic drag coefficient and proton conductivity in Nafion® membrane for PEMFC. Int. J. Hydrog. Energy.

[34] Jiang B., Wu L., Yu L., Qiu X., Xi J. (2016). A comparative study of Nafion series membranes for vanadium redox flow batteries. J. Membr. Sci..

[35] Mu D., Yu L., Liu L., Xi J. (2017). Rice paper reinforced sulfonated poly(ether ether ketone) as low–cost membrane for vanadium flow batteries. ACS Sustain. Chem. Eng..

[36] Zakil F.A., Kamarudin S.K., Basri S. (2016). Modified Nafion membranes for direct alcohol fuel cells: An overview. Renew. Sustain. Energy Rev..

[37] Ghasemi M., Daud W.R.W., Alam J., Jafari Y., Sedighi M., Aljlil S.A., IIbeygi H. (2016). Sulfonated poly ether ether ketone with different degree of sulphonation in microbial fuel cell: Application study and economical analysis. Int. J. Hydrog. Energy.

[38] Wei X., Nie Z., Luo Q., Li B., Sprenkle V., Wang W. (2013). Polyvinyl chloride/silica nanoporous composite separator for all-vanadium redox flow battery applications. J. Electrochem. Soc..

[39] IAEA (2004). Advances in Radiation Chemistry of Polymers.

[40] Li X., dos Santos A.R., Drache M., Ke X., Gohs U., Turek T., Becker M., Kunz U., Beuermann S. (2017). Polymer electrolyte membranes prepared by pre-irradiation induced graft copolymerization on ETFE for vanadium redox flow battery applications. J. Membr. Sci..

[41] Li X. (2016). Synthese und Charakterisierung von Polymer–Elektrolyt–Membranen für die Anwendung in Brennstoffzellen und Vanadium–Redox–Flow–Batterien. Ph.D. Thesis.

[42] Zukowska G., Williams J., Stevens J.R., Jeffrey K.R., Lewera A., Kulesza P.J. (2004). The application of acrylic monomers with acidic groups to the synthesis of proton-conducting polymer gels. Solid State Ion..

[43] Kye H., Koh Y.G.V., Kim Y., Han S.G., Lee H., Lee W. (2017). Tunable temperature response of a thermochromic photonic gel sensor containing N-Isopropylacrylamide and 4-Acryloyilmorpholine. Sensors.

[44] Drozdov A.D., Sanporean C.G., Christiansen J.D.C. (2015). Mechanical response of HEMA gel under cyclic deformation: Viscoplasticity and swelling–induced recovery. Int. J. Solids Struct..

[45] Santander-Borrego M., Green D.W., Chirila T.V., Whittaker A.K., Blakey I. (2014). Click functionalization of methacrylate-based hydrogels and their cellular response. J. Polym. Sci. Part A Polym. Chem..

[46] Schmidt C., Schmidt-Naake G. (2007). Proton conducting membranes obtained by doping radiation–grafted basic membrane matrices with phosphoric acid. Macromol. Mater. Eng..

[47] Schmidt-Naake G. (2009). Polymer-Säure-Komposite auf Basis strahlungsinduzierter Pfropfpolymerisation für Hochtemperatur-PEM-Brennstoffzellen. Chem. Ing. Technol..

[48] Schmidt C., Schmidt-Naake G. (2008). Phosphorsäuredotierte protonenleiter auf basis von aminierten membranen aus ETFE-graft-poly-(glycidylmethacrylat)-derivaten. Chem. Ing. Technol..

[49] Schmidt C., Glück T., Schmidt-Naake G. (2007). Proton exchange membranes by radiation-induced graft polymerization of glycidyl methacrylate on poly(tetrafluoroethylene-alt-ethylene) (ETFE)-modification with butyl acrylate and acrylonitrile. Chem. Ing. Technol..

[50] Chandan A., Hattenberger M., EI-kharouf A., Du S., Dhir A., Self V., Pollet B.G., Ingram A., Bujalski W. (2013). High temperature (HT) polymer electrolyte membrane fuel cells (PEMFC)—A review. J. Power Sources.

[51] Beuermann S., Buback M. (2002). Rate coefficients of free-radical polymerization deduced from pulsed laser experiments. Prog. Polym. Sci..

[52] Nikolic G., Zlatkovic S., Cakic M., Cakic S., Lacnjevac C., Rajic Z. (2010). Fast fourier transform IR characterization of epoxy GY systems crosslinked with aliphatic and cycloaliphatic EH polyamine adducts. Sensors.

[53] Nafion N115, N117, N1110, Ion Exchange Materials, Extrusion Cast Membranes. https://www.chemours.com/Nafion/en_US/assets/downloads/nafion-extrusion-cast-membranes-product-information.pdf..

