# Supplementary material for: Preparation of Polymer Electrolyte Membranes via Radiation-Induced Graft Copolymerization on Poly(ethylene-alt-tetrafluoroethylene) (ETFE) Using the Crosslinker N,N′-Methylenebis(acrylamide)
Source: Membranes (Basel). 2018 Nov 6;8(4):102. doi: 10.3390/membranes8040102 (PMC6316420; doi:10.3390/membranes8040102)
Supplement: Supplementary file 1 [file membranes-08-00102-s001.pdf]

Supplementary Materials

Preparation of Polymer Electrolyte Membranes via Radiation-Induced Graft Copolymerization on Poly(ethylene-alt-tetrafluoroethylene) (ETFE) Using the Crosslinker *N,N′*-Methylenebis(acrylamide)

Xi Ke [1], Marco Drache [1], Uwe Gohs [2], Ulrich Kunz [3,4] and Sabine Beuermann [1,*]

1 Institute of Technical Chemistry, Clausthal University of Technology, Arnold-Sommerfeld-Straße 4, 38678 Clausthal-Zellerfeld, Germany; xi.ke@tu-clausthal.de (X.K.); marco.drache@tu-clausthal.de (M.D.)
2 Leibniz Institute of Polymer Research, Hohe Straße 6, 01069 Dresden, Germany; gohs@ipfdd.de
3 Institute of Chemical and Electrochemical Process Engineering, Clausthal University of Technology, Leibnizstraße 17, 38678 Clausthal-Zellerfeld, Germany; kunz@icvt.tu-clausthal.de
4 Energie-Forschungszentrum Niedersachsen, Am Stollen 19A, 38640 Goslar, Germany
* Correspondence: sabine.beuermann@tu-clausthal.de; Tel.: +49-532-372036

Design of Experiment

The statistical experimental design attempts to find correlations between the influencing variables (here: reaction temperature, solvent fraction, dose) and the target variable (here: grafting degree). The goal is to find the PEM preparation conditions for maximum grafting degree. In this work, the parameters are systematically varied with the use of central composite design (CCD). This method consists of a cube containing 2^3 points, a central point and a cross through it, resulting in points on each center of the area. Here the 2 stands for the steps from the starting point, i.e. the level of variations for a parameter. The exponent 3 stands for the number of influencing variables. The starting point and the step size of the experimental design are described by Table S1. Table S2 provides an overview of the different reaction conditions of the original plan.

Table S1. Starting point (x_{io}) and the step size (dx_i) of the influencing variables.

	Temperature *T*/°C	Solvent Fraction/%	Dose/kGy
x_i	x_1	x_2	x_3
x_{io}	80	50	50
dx_i	20	10	10

Table S2. Points of the experimental design and target value *DG*.

No.	x_1	x_2	x_3	*T*/°C	Solvent Fraction/%	Dose/kGy	*DG*/%
# 1	−1	−1	1	60	40	60	234
# 2	−1	−1	−1	60	40	40	284
# 3	−1	1	1	60	60	60	182
# 4	−1	1	−1	60	60	40	244
# 5	0	0	0	80	50	50	230
# 6	1	0	0	100	50	50	160
# 7	0	−1	0	80	40	50	193
# 8	0	0	−1	80	50	40	90
# 9	0	0	1	80	50	60	94
# 10	−1	0	0	60	50	50	291
# 11	1	−1	−1	100	40	40	77

# 12	1	−1	1	100	40	60	134
# 13	1	1	1	100	60	60	61
# 14	1	1	−1	100	60	40	56
# 15	0	1	0	80	60	50	89

To get an improved understanding of the correlation between *DG* and all three influencing parameters, additional points that do not belong to the CCD were also studied. These points are shown in Table S3.

Table S3. Additional points to the experimental plan and target value *DG*.

No.	x_1	x_2	x_3	*T*/°C	Solvent Fraction/%	Dose/kGy	*DG*/%
# 16	−1	1	0	60	60	50	84
# 17	0	1	1	80	60	60	78
# 18	0	1	−1	80	60	40	72
# 19	−1	0	1	60	50	60	392
# 20	0	−1	−1	80	40	40	175
# 21	0	−1	1	80	40	60	218
# 22	−1	0	−1	60	50	40	361
# 23	1	0	−1	100	50	40	72
# 24	−1	−1	0	60	40	50	43
# 25	1	0	1	100	50	60	81
# 26	1	−1	0	100	40	50	89
# 27	1	1	0	100	60	50	74

The individual experiments may be represented in a three-dimensional cubic diagram. In a central composite design, there is distinction between different spheres, which can be explained well using Figure S1. The assignment of the points to the spheres results from the ascending distance of the spheres from the central points. The 0th sphere is the central point. The first sphere corresponds to the 6 points in surface centers. Sphere 2 contains the points on the bisector. The eight vertices belong to 3rd spheres.

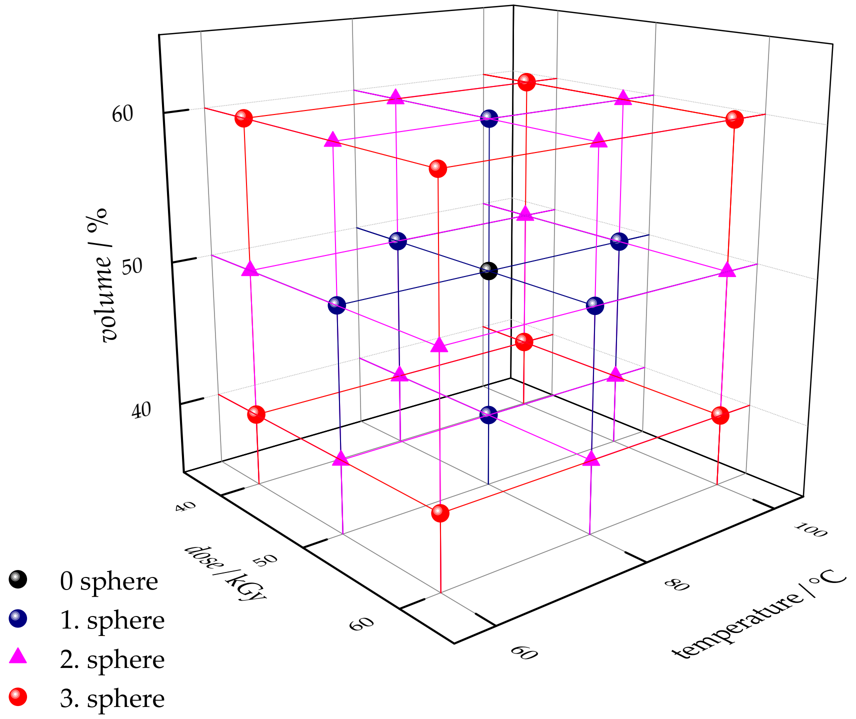

Figure S1. Three-dimensional representation of the experimental design.

With this design the interesting experimental area can be systematically investigated. The desired reaction time for these points is 3 hours. In fact, this is not practicable for all the experiments. There are two limited criteria, namely a strong increase in the viscosity of the solution and the increased formation of polymer chunks in the solvent. Both effects occur not only individually but also together. For a clear evaluation, the actual reaction time for all the points in the experimental design is illustrated in Figure S2 (identical to Figure 3 of the main text).

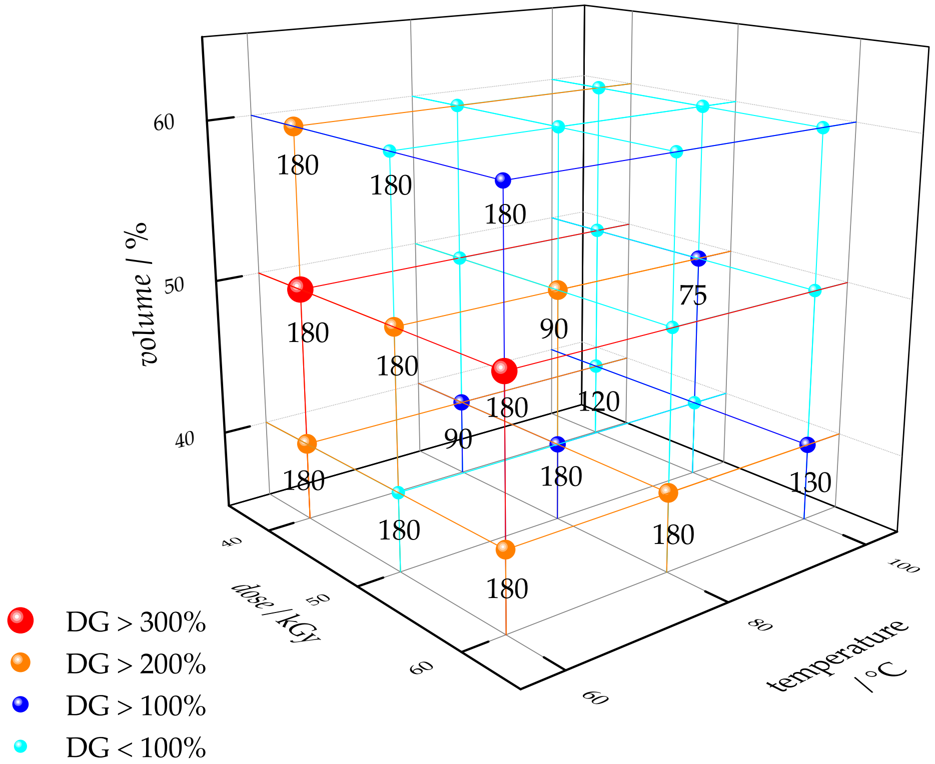

Figure S2. Applied reaction times in minutes with appropriate *DG* in the experimental design.
